# A179 AUTOLOGOUS BONE MARROW TRANSPLANT FOR REFRACTORY CROHN’S DISEASE: A CASE SERIES

**DOI:** 10.1093/jcag/gwab049.178

**Published:** 2022-02-21

**Authors:** J Buttar, N H Akhtar, D Akhtar, C Barker, B Bressler, K Atkinson

**Affiliations:** 1 The University of British Columbia Faculty of Medicine, Vancouver, BC, Canada; 2 Medicine, The University of British Columbia Faculty of Medicine, Vancouver, BC, Canada; 3 Pacific Gastroenterology Associates, Vancouver, BC, Canada; 4 Royal Columbian Hospital, New Westminster, BC, Canada; 5 BC Children’s Hospital, Vancouver, BC, Canada; 6 School of Medicine, University College Dublin, Dublin, Ireland

## Abstract

**Background:**

Crohn’s disease (CD), a form of inflammatory bowel disease (IBD) is a chronic, immune mediated condition characterized by gastrointestinal inflammation. Approximately 25% of CD patients have pharmacologically refractory disease, in which stem cell therapy has been shown to play a role.

**Aims:**

A case series was performed to analyze the efficacy of autologous bone marrow transplantation (ABMT) for refractory CD in British Columbia(B.C).

**Methods:**

A chart review was conducted on patients who had undergone ABMT for treatment refractory CD between 2001 to 2021 in B.C. Demographic, clinical, laboratory and endoscopic data was collected.

**Results:**

Case details are summarized in Table 1. 3 patients(2 female and one male) were included. All patients failed conventional therapies prior to ABMT. 2 patients underwent surgical intervention (colectomy with ileostomy) prior to ABMT. Average time from diagnosis to ABMT was 8.83 + 6.6 years. All 3 patients received standard myeloablative therapy. There were no intestinal complications post ABMT. 6 months post-ABMT transplant, all 3 patients showed significant improvement, with CDAI scores <150. Endoscopic assessment post-ABMT revealed endoscopic remission in 2 of the 3 patients. 2 of the 3 patients were in clinical remission at 12 months follow up. 1 patient relapsed and required further immunosuppressive therapy. This patient was trialed on thalidomide at 15 months post-ABMT and ultimately passed away 18 months post-ABMT from an unrelated cause. 10 years post-transplant, the remaining 2 patients remain in clinical and endoscopic remission with CDAI scores <150.

**Conclusions:**

Despite medical and surgical therapeutic advances, a subset of CD patients develop refractive disease associated with significant morbidity and mortality. In this population, there is increasing evidence in support of stem cell therapy as a treatment modality, with acute mortality less than 5% for patients with malignancy driven primarily by infectious complications and treatment-related toxicity. Clinical trials are currently underway to evaluate ABMT in CD. This case series presents the only Canadian data to date on the use of ABMT for refractory CDs and their subsequent follow up.

**Funding Agencies:**

None

**Table:** Summary of Patient Demographics, Clinical and Disease Characteristics

	Case 1	Case 2	Case 3
Sex	M	F	F
Year of Birth	1983	1992	2000
Year of Diagnosis	1994	2005	2011
Extraintestinal Manifestations of Crohn’s Disease	Osteoporosis	Arthritis	Arthritis
Previous Treatments	5-ASA, Corticosteroids, Azathioprine, Infliximab, Adalimumab	5-ASA, Corticosteroids, Methotrexate, Infliximab, Adalimumab	Corticosteroids, Azathioprine, Infliximab, Visiluzumab, Adalimumab, Thalidomide
Surgical History	Total Colectomy and Small Bowel Resection	None	Total Colectomy
Age at Stem Cell Therapy	30	18	14
Recurrence of IBD after stem cell therapy	No	No	Yes
Follow up after stem cell therapy	8 years	10 years	1.5 years
Endoscopic control after transplantation	2014: EGD showed pristine mucosa, SES score of 0	2019: Lower GI endoscopy revealed completely normal colonic mucosa with SES score of 0.	2015: Upper GI endoscopy showed mild patchy erythema involving gastric body. No ulcers. Patchy duodenitis with small aphthous ulcers. 2015: Lower GI endoscopy demonstrated patchy erosions in rectum, normal ileum and colonic mucosa.

